# Evaluation of antimicrobial usage in companion animals at a Veterinary Teaching Hospital in Nigeria

**DOI:** 10.1038/s41598-023-44485-w

**Published:** 2023-10-24

**Authors:** O. O. Adebowale, A. B. Jimoh, O. O. Adebayo, A. A. Alamu, A. I. Adeleye, O. G. Fasanmi, M. Olasoju, P. O. Olagunju, F. O. Fasina

**Affiliations:** 1 Department of Veterinary Public Health and Preventive Medicine, College of Veterinary Medicine, Federal University of Agriculture Abeokuta, Abeokuta, Ogun State Nigeria; 2 Veterinary Teaching Hospital, College of Veterinary Medicine, Federal University of Agriculture Abeokuta, Abeokuta, Ogun State Nigeria; 3 Department of Veterinary Medicine, College of Veterinary Medicine, Federal University of Agriculture Abeokuta, Abeokuta, Ogun State Nigeria; 4 Department of Veterinary Laboratory Technology, Federal College of Animal Health and Production Technology, Ibadan, Oyo State Nigeria; 5 ECTAD, Food and Agriculture Organization of the United Nations (FAO), Rome, Italy; 6 Department of Veterinary Tropical Diseases, University of Pretoria, Pretoria, South Africa

**Keywords:** Health care, Medical research

## Abstract

This study investigated various qualitative and quantitative indices of antimicrobial use (AMU) in companion animals (CAs) at a Veterinary Teaching Hospital (VTH-A) and its annex (VTH- B) from 2019 to 2021. For 694 documented animals, antimicrobial administrations (AADs) were 5, 278 times, of which 98.8% (5217) and 1.2% (61) were in dogs and cats respectively. At the VTH- A, oxytetracycline (1185 times, 22.5%) was mostly administered in dogs and metronidazole (26 times, 0.5%) in cats. Similarly, at VTH- B, oxytetracycline was administered 895 times (17.0%) in dogs while amoxicillin was given 7 times (0.1%) in cats. The prescription diversity (PD) was estimated at 0.73 and 0.82 in VTH-A and VTH-B respectively. The quantity of antimicrobials (AMs) used was 10.1 kg (A, 6.2 kg and B, 3.9 kg). Oxytetracycline administrations and quantity of metronidazole (P < 0.0001) were higher than other Active Ingredients (AIs). Furthermore, 16.5% of AIs were classified as Critically Important Antibiotics (CIA) with the highest priority, while enrofloxacin, ciprofloxacin, and azithromycin fell under the World Health Organisation (WHO) Watch group. The In-Depth Interview (IDI) indicated that the high frequency of oxytetracycline administrations was linked with being the first choice for blood parasite treatment by the clinicians at the hospital. The quantity of metronidazole used was perceived to be higher due to the clinicians' preference for the treatment of acute gastroenteritis, its wider dose range, and the frequency of administration (bi-daily). The study provides baseline data on AMU indices in CAs, for the development of antimicrobial stewardship (AMS) and communication training, and policy modifications to enhance antimicrobial therapy optimization in tertiary veterinary hospital care in Nigeria.

## Introduction

University Veterinary Teaching Hospitals (VTHs) are paramount in clinical teaching in veterinary medicine and have existed since the early twentieth century^1^. The role of a Veterinary Teaching Hospital includes the (1) training of future professional and empirical veterinarians through teaching and clinical education, (2) societal contribution of veterinary medical practice in the region where it is based; and (3) conducting research, development, and validation of advanced veterinary medicine among others^1,2^. The VTHs deal with livestock and companion animals (CAs), game and aquatic animals, as well as rare species.

Antimicrobials (AMs) are crucial for the protection of human health, animal health, and welfare. However, the indiscriminate use of AMs due to unrestricted access and weak regulatory policies in medical and veterinary sectors are potential drivers for Antimicrobial Resistance (AMR) in developing countries^3–7^. In CAs, antimicrobial prescription and the impact on AMR have been neglected despite the large amount of evidence of cross-species transfer of resistant bacteria^8,9^. Moreover, CAs share some classes of AMs including medically important ones, which could promote the development and spread of AMR relevant to human health^10^. As a consequence, addressing antibiotic misuse and overuse, particularly those deemed to be of the highest priority critically importance (HPCIAs) in CAs, is evolving to becoming of “One Health” interest^11,12^.

The WHO classified antimicrobials into Access, Watch, and Reserve groups (AWaRe;^13^) to promote prioritization, and surveillance of the HPCIAs and support Antibiotic Stewardship (AMS) efforts at local, national, and global levels. However, little is known qualitatively and quantitatively about AMs used, particularly in the tertiary veterinary care hospitals in Nigeria. In general, AMU is rarely tracked in veterinary institutions mainly due to a lack of digitalization of medical records to generate AMU data^14^. Therefore, the study evaluated and characterized AMs used at a tertiary care animal teaching hospital using metrics such as the administered daily amount (ADA) and prescription diversity (PD). In addition, we categorized AMs based on WHO critically important ones in human medicine and AWaRe (Access, Watch, Reserve), and conducted in-depth focus group interviews among clinicians of the hospital to investigate their experiences and perceptions around AMU and AMR. Acquiring this data is critical in identifying context—specific interventions to promote the optimization of antimicrobials in CAs.

## Materials and methods

### Study setting

We selected a veterinary teaching hospital and its satellite facility (annex) for this study. The VTH serviced a city in South West Nigeria with a human population of approximately 571,500 people (https://worldpopulationreview.com/world-cities/abeokuta-population). Nigeria is a sub-Saharan West African country with well over 10 million pet dogs^15^. The hospital under study has an annex and both serve as a referral center for private and government veterinary clinics^16^. The hospital offers services to all varieties of animals such as CAs, food animals, and wildlife, and has board-certified veterinarians who prescribe medications including AMs for various empirical use in animals. Hence, clinical routine data from the hospital can be used to evaluate AMU patterns in CAs, especially dogs and cats. The main VTH was designated VTH-A and the annex was designated VTH-B for this study.

### Study design

A mixed methods assessment was conducted as follows:

(1) A 3-year retrospective analysis of medical records on AMU from the veterinary tertiary hospital and its annex from 2019 to 2021 was conducted. For this study, AMs were considered “both synthetic and non-synthetic”. (2) An in-depth interview (IDI) was held among the antimicrobial prescribing clinicians (APCs) at the VTH to explore their perceptions towards rational antimicrobial prescriptions, drivers for AMs prescriptions or choices, and AMR. All qualified veterinarians prescribing antibiotics were eligible to participate in the discussion. The interview guide was developed based on the outcomes of analysis of patients’ medical records and previous research (Limato et al. 2022), and validated by qualitative research experts.

### Data collection and management

For this study, we assumed that all billed drugs were used to treat pets. Information obtained included the patient’s case file I.D., sex, breed, species, age, weight at treatment, antimicrobial prescription, and patient category (out-patient, in-patient, or in-house point-of-care administration). For the drugs, data were generated on the total number of antimicrobials administered; treatment date, active ingredients, amount and unit of the preparation; and whether the drug was administered during the visit or dispensed to the owner; whether treatment plans were completed, and route of drug administration. Additionally, the active ingredients in each AM were classified according to the WHO-CIA and AWaRe.

#### Inclusion criteria

Only dogs and cats that were prescribed at least one antimicrobial drug within the study period were considered for inclusion in the study.

#### Exclusion criteria

Dogs and cats whose files did not have adequate documentation on active ingredients, amount and units of preparation, and at least a veterinarian certifying treatment plans or protocols were excluded from the study. All other animal species were also excluded from the study because the overwhelming number of animal species presenting at the VTHs were dogs and cats.

### In-depth interview

The IDI explored results from the retrospective analysis of AMU in dogs and cats from 2019 to 2021. Before the interviews, potential participants were sent invitations and consent forms. Once the participants had signed the form, he or she was enrolled in the discussion. The date and location were agreed on by the interviewers and participants. The interview was continuous and follow-up questions were asked based on the responses of participants. The discussion was in English, audio-recorded, and transcribed verbatim. The IDI questions and guidelines were reviewed by two experts in the field of qualitative research. No personal data from veterinary practitioners were recorded.

The subject of the IDI was grouped into four themes, which addressed AMU practices, drivers, and challenges; Infection control practices; awareness about WHO-CIA and AWaRe; and opinions about AMR and control in veterinary hospitals.

### Statistical analyses

Data generated were captured and merged into Microsoft Excel, 2016 (Microsoft Corporation, Redmond, WA) and transferred to Statistical Package for the Social Science version 23.0 (IBM SPSS 23.0, USA) for both descriptive and inferential statistics. The age and weight of patients were subjected to normality testing using Kolmogorov–Smirnov (> 0.05), which informed our use of median or Mean ± SD. Data on antibiotic classes and active ingredient administrations were analysed descriptively as proportions and percentages. According to Schnepf et al.^8,17^, the calculation of the Administered Daily Amount (ADA) was evaluated using the formula:

- **Administered Daily Amount (ADA)** = amount of drug × proportion of active ingredient in the drug
- **Prescription Diversity (PD)** = 
  $$1 - \frac{{\sum {np(np - 1)} }}{NP(NP - 1)}$$

Where np = number of prescriptions of a particular pharmaceutical class (PC) within a PF.

NP = the total number of prescriptions within a PF.

*Prescription diversity (PD) is defined as “the frequency and variety with which a practice prescribes pharmaceutical classes (PC) within a determined pharmaceutical family (PF)” *^18^.

Further comparisons were performed using ordinary—two-way ANOVA and Dunnett's post hoc analyses to test the mean variation in the number of drug administrations and quantity at VTH -A and VTH-B. These comparisons were made only for data retrieved from dogs. Records for cats were few and unanalysable.

### Ethical approval

This study was performed in line with the principles of the Declaration of Helsinki^19^. Ethical Approval (Reference number FUNAAB/COLVET/CREC/2021/10/01) was obtained from the College of Veterinary Medicine Research Ethics (CREC), Federal University of Agriculture Abeokuta, Ogun State Nigeria.

### Informed consent

Informed and signed consent was obtained from the VTH’s Director to have access to patient files from 2019 to 2021 and to conduct interviews among APCs. Similarly, signed consent was obtained from APCs before the interview commenced. Permission to make audio recordings of the discussions was obtained and the clinicians were aware participation in the discussion was voluntary. Personal identifiers were not collected and information provided by participants was treated confidentially. Every participant was aware of his/her right to discontinue participation at any stage of the study according to the World Medical Association Declaration of Helsinki, 2001. Only authorized persons (the research team) had access to the hospital information used for this research, and none were allowed to keep a personal copy of the records.

## Results

### Characteristics of companion animals (Dogs and Cats) documented in files at the Veterinary Teaching Hospital (A&B), Abeokuta, Nigeria from 2019 to 2021

Detailed data were collected for a total of 694 animals comprising 684 dogs (98.6%) and 10 cats (1.4%). The files of 324 (46.7%) and 370 (53.3%) animals were obtained from VTH-A and B respectively (Table 1). 692 animals were documented as outpatients (99.7%). The animals’ median age was 4 months (minimum 1 month, maximum 12 years 3 months), and weight was 10 kg (minimum 4 kg, maximum 101.1 kg). Common dog breeds were Alsatian/German Shepherd (259, 37.3%), Boerboel (115, 16.6%), Rottweiler (68, 9.8%), and Lhasa Apso (39, 5.4%).

**Table 1:** Companion animal information retrieved from VTH Records, 2019–2021.

Variables (n = 694)	Category	Frequency (%)	95% CI
Age (months)	≤ 4 months	351 (50.6)	46.7–54.4
	> 4 months	343 (49.4)	46.0–53.1
Sex	Male	324(46.7)	43.0–50.4
	Female	370 (53.7)	49.5–57.1
Weight (Kg)	≤ 10	352 (50.7)	47.0–54.4
	> 10	342 (49.3)	45.5–53.1
Species	Canine	684 (98.6)	97.3–99.3
	Feline	10 (1.4)	0.7–2.7
Breed	Alsatian/German Shepherd	259 (37.5)	33.8–51.0
	Rottweiler	68 (9.8)	7.8–12.3
	Boerboel	115 (16.6)	14.0–19.5
	Caucasian	57 (8.2)	6.4–10.5
	Lhasa Apso	39 (5.6)	4.1–7.6
	Doberman	10 (1.4)	0.7–2.7
	Local/Mongrel	31 (4.5)	3.1–6.3
	Samoyed	5 (0.7)	0.3–1.7
	Italian Mastiff	1 (0.1)	< 0.01–0.9
	Pitbull	27 (3.9)	2.7–5.6
	American Eskimo	39 (5.6)	4.1–7.6
	Great Dane	3 (0.4)	0.08–1.3
	Terrier cross	1 (0.1)	< 0.01–0.9
	Cane Terrier	3 (0.4)	0.08–1.3
	Cane Cursor	2 (0.3)	< 0.01–0.9
	Chow Chow	10 (1.4)	0.7–2.7
	Bull Mastiff	3 (0.4)	0.08–1.3
	Pug	4 (0.6)	0.16–1.5
	Labrador	3 (0.4)	0.08–1.3
	St Bernard	1 (0.1)	< 0.01–0.9
	Belgian Maltese	1 (0.1)	< 0.01–0.9
	Siberian Husky	2 (0.3)	< 0.01–0.9
	Local (cats)	10 (1.4)	0.7–2.7

### Antimicrobial use and prescription diversity in dogs and cats from 2019 to 2021 (VTH-A )

**Figure 1:**
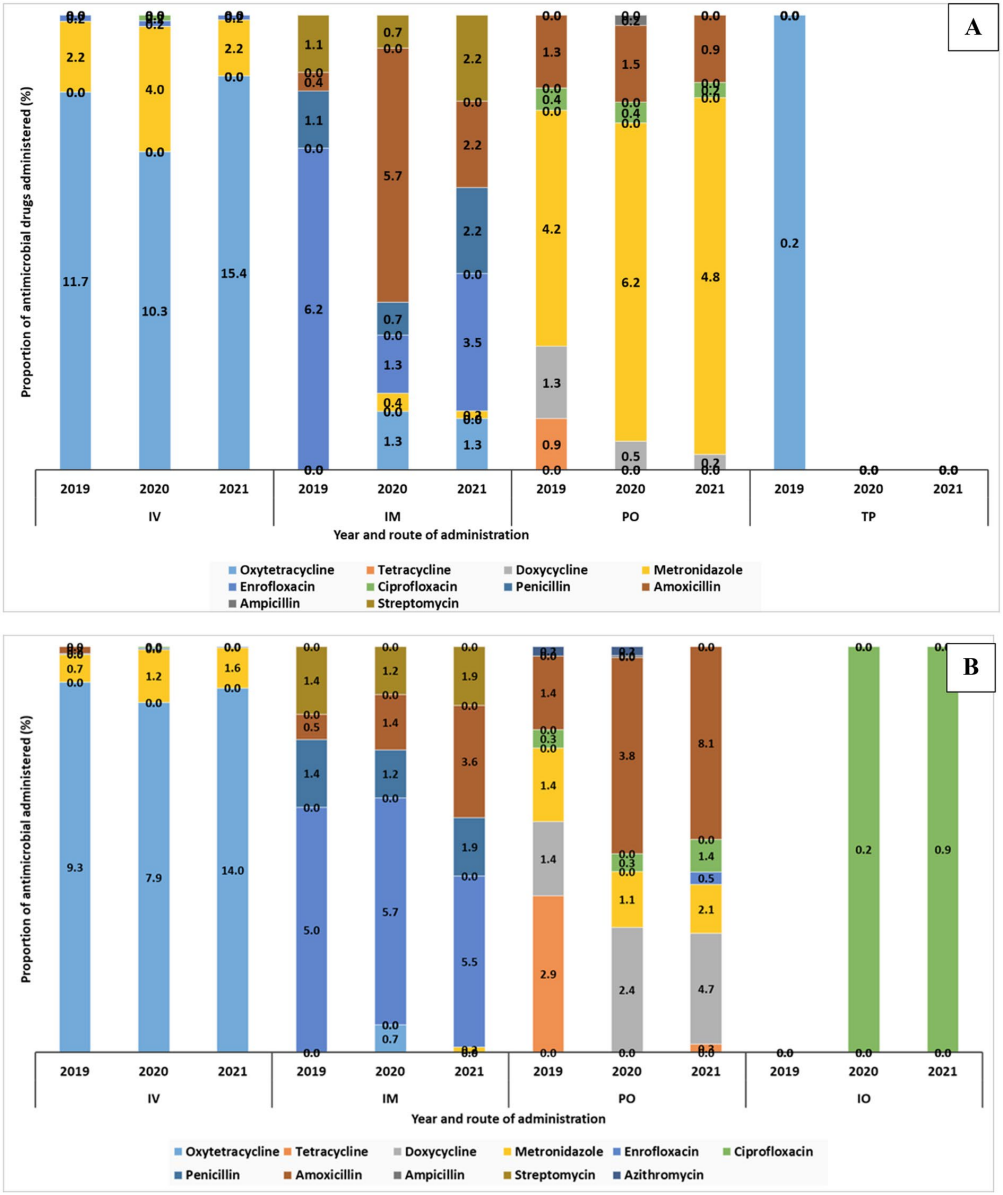
The various antimicrobial active ingredients and routes of administration for dogs and cats at the (**A**). VTH-A and (**B**). VTH-B, from 2019  to 2021. IV, Intravenous; IM, intramuscular; PO, Oral or Per Os; IO, intra-ocular and TP, topical.

Two thousand five hundred and six (2506 antimicrobial administrations (AMAs) were documented, of which 2452 (97.8%) were in dogs and 54 (2.2%) in cats. The outpatient point of care accounted for all AMA (100.0%). The main routes of drug administrations in the descending order were intravenous (IV, 46.5%), intramuscular injections (IM, 30.4%), oral (PO, 22.9%), and topical (TP, 0.2%). Oxytetracycline (37.4%), enrofloxacin (11.0%) and metronidazole (15.2%) were most administered IV, IM, and PO respectively. Figure 1A illustrates the various AIs and routes of administration at VTH-A. Figure 1B is for VTH-B, which is described later on.

The common ACs and AIs used for dogs in the descending order were tetracyclines (oxytetracycline, 48.3%), Nitroimidazoles (metronidazole 17.7%), Quinolones (enrofloxacin, 9.3%), and Penicillins (amoxicillin, 8.3%). For cats, Nitroimidazoles (metronidazole 48.1%), Tetracyclines (oxytetracycline, 14.8%), and Penicillins (amoxicillin, 9.3%) were common (Table 2). Out of the 324 files, 313 (96.6%) CAs completed AM drug prescriptions (305 dogs and 8 cats). Metronidazole, amoxicillin, ciprofloxacin, doxycycline, and tetracycline tablets were main AMs dispensed to dogs (13/313, 4.2%) for subsequent administration at home by pet owners. No records of follow-up were observed.

**Table 2:** Number of antimicrobial active ingredients administered in dogs and cats in 2019, 2020, and 2021documented at the VTH-A.

Antimicrobial class and active ingredients	Dogs					Cats				
	2019	2020	2021	Total administrations documented (TAD)	TAD (%)	2019	2020	2021	Total administrations documented (TAD)	TAD (%)
Tetracyclines	**487**	**311**	**509**	**1307**	**53.3**	**8**	**0**	**0**	**8**	**14.8**
Oxytetracycline	388	293	504	1185	48.3	8	0	0	8	14.8
Doxycycline	75	18	5	98	4.0	0	0	0	0	0.0
Tetracycline	24	0	0	24	1.0	0	0	0	0	0.0
Penicillins	**73**	**127**	**118**	**318**	**13.0**	**0**	**5**	**5**	**10**	**18.5**
Penicillin	28	29	55	112	4.6	0	0	5	5	9.3
Amoxicillin	45	96	63	204	8.3	0	5	0	5	9.3
Ampicillin	0	2	0	2	0.1	0	0	0	0	0.0
Aminoglycosides	**28**	**29**	**55**	**112**	**4.6**	**0**	**0**	**5**	**5**	**9.3**
Streptomycin	28	29	55	112	4.6	0	0	5	5	9.3
Macrolides	**0**	**0**	**0**	**0**	**0.0**	**0**	**0**	**0**	**0**	**0.0**
Azithromycin	0	0	0	0	0.0	0	0	0	0	0.0
Imidazoles	**118**	**176**	**141**	**435**	**17.7**	**11**	**15**	**0**	**26**	**48.1**
Metronidazole	118	176	141	435	17.7	11	15	0	26	48.1
Quinolones	**108**	**54**	**118**	**280**	**11.4**	**0**	**0**	**5**	**5**	**9.3**
Enrofloxacin	73	41	113	227	9.2	0	0	0	0	0.0
Ciprofloxacin	35	13	5	53	2.2	0	0	5	5	9.3
Total	**814**	**697**	**941**	**2452**	**100.0**	**19**	**20**	**15**	**54**	**100.0**

Bold-Summary of drug administration per antimicrobial group, the corresponding active ingredients are indicated below. 0 observed zero i.e. not administered during the period of study.

**Figure 2:**
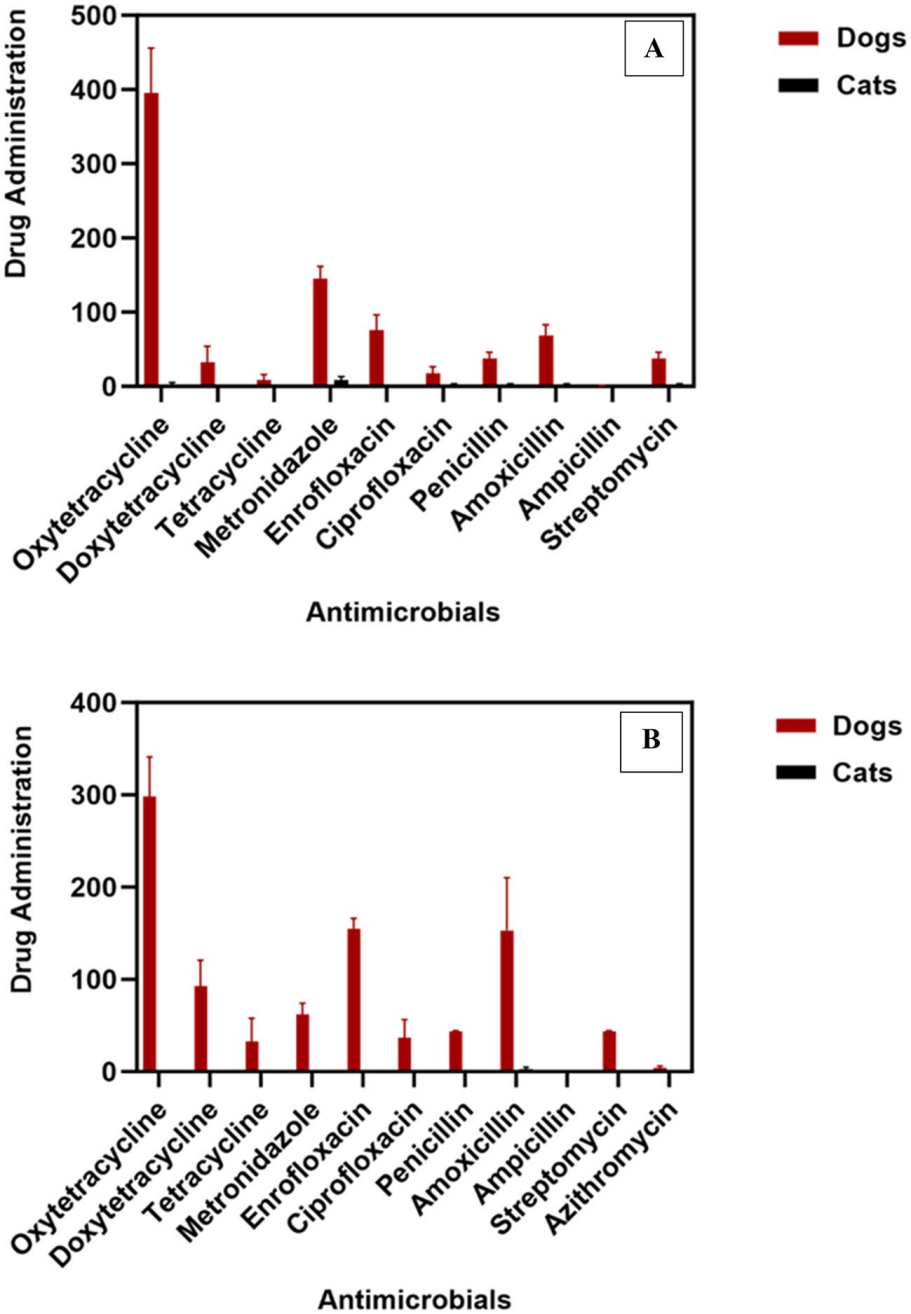
The mean number of antimicrobial administrations from 2019 to 2021 for (**A**) VTH-A and (**B**) VTH-B. Error bars are SEM of data sets for three years.

Oxytetracycline was administered 1185 times and followed by metronidazole (435 times) in dogs. The prescription diversity (PD) of AMs used in 2019, 2020, and 2021 were 0.73, 0.73, and 0.67 respectively with 1.0 representing maximal richness and diversity. For cats, metronidazole was most prescribed (26 times; Table 2), and PD for AMAs were 0.49, 0.38, and 0.68 in 2019, 2020, and 2021 respectively. The number of administrations for each AIs for over 3 years is presented in Fig. 2A.

### Antimicrobial use and prescription diversity in dogs and cats from 2019 to 2021 (VTH – B)

2772 AMAs were documented from 2019 to 2021 of which 2765 (99.7%) were in dogs’ and 7 (0.3%) in cats’ treatments. A higher number of AMAs was documented for outpatient care (99.8%), while 0.2% accounted for inpatients.

The common routes of drug administration were intravenous (34.9%), oral (32.5%), intramuscular injections (31.6%), and Intraocular (1.0%). Oxytetracycline (31.3%), enrofloxacin (16.2%), and amoxicillin (13.3%) were most frequently administered IV, IM, and PO drugs respectively. Figure 1B above described the various active ingredients (AIs) and routes of administration. For dogs in the decreasing order, tetracyclines (oxytetracycline, 32.4%), Quinolones (enrofloxacin, 16.8%), and Penicillins (amoxicillin, 16.6%). This was similar to what was documented at VTH- A. In contrast, Penicillins (amoxicillin, 100.0%) were the only AMs used in cats during the study period. Antimicrobial prescriptions were completed in 90.2% of the 370 patients. Metronidazole, amoxicillin, ciprofloxacin, doxycycline, oxytetracycline, tetracyclines, enrofloxacin, and azithromycin tablets were main AMs dispensed for patients especially dogs (41/332, 12.3%) for subsequent administration at home by pet owners. No records of follow-up on pet owners were observed.

For dogs, oxytetracyclines were administered 895 times, followed by enrofloxacin (459 times) and amoxicillin (465 times). The least administered AMs were the Macrolides (azithromycin, 12 times). The number of drug administrations for each AI over 3 years is illustrated in Fig. 2B. The PD for dogs in 2019, 2020, and 2021 were 0.79, 0.82, and 0.81 respectively. According to the data, no antimicrobial drug administration was documented in 2019 and 2020 for cats. The PD was estimated at 0.43 in 2021. Table 3 describes the total drug administrations in dogs and cats at VTH-B. Overall there was no significant variation in the number of drug administrations at the VTH, A, and B (P = 0.39). However, oxytetracycline administration was higher (P < 0.0001) than other AIs used in dogs.

**Table 3:** Number of antimicrobial active ingredients administered in dogs and cats in 2019, 2020, and 2021 documented at VTH– B.

Antimicrobial class and active ingredients	Dogs					Cats				
	2019	2020	2021	Total administrations documented (TAD)	(TAD)%	2019	2020	2021	Total administrations documented (TAD)	**(TAD)%**
Tetracyclines	**386**	**332**	**556**	**1274**	**46.1**	**0**	**0**	**0**	**0**	**0.0**
Oxytetracycline	273	240	382	895	32.4	0	0	0	0	0.0
Doxycycline	47	87	144	278	10.1	0	0	0	0	0.0
Tetracycline	83	5	10	98	3.5	0	0	0	0	0.0
Penicillins	**107**	**175**	**312**	**594**	**21.5**	**0**	**0**	**7**	**7**	**100.0**
Penicillin	44	41	45	130	4.7	0	0	0	0	0.0
Amoxicillin	65	134	260	459	16.6	0	0	7	7	100.0
Ampicillin	0	0	0	0	0.0	0	0	0	0	0.0
Aminoglycosides	**44**	**41**	**45**	**130**	**4.7**	**0**	**0**	**0**	**0**	**0.0**
Streptomycin	44	41	45	130	4.7	0	0	0	0	0.0
Macrolides	**5**	**7**	**0**	**12**	**0.4**	**0**	**0**	**0**	**0**	**0.0**
Azithromycin	5	7	0	12	0.4	0	0	0	0	0.0
Imidazoles	**45**	**54**	**86**	**185**	**6.7**	**0**	**0**	**0**	**0**	**0.0**
Metronidazole	45	54	86	185	6.7	0	0	0	0	0.0
Quinolones	**142**	**177**	**251**	**570**	**20.6**	**0**	**0**	**0**	**0**	**0.0**
Enrofloxacin	137	153	175	465	16.8	0	0	0	0	0.0
Ciprofloxacin	9	24	76	109	3.9	0	0	0	0	0.0
Total	**729**	**786**	**1250**	**2765**	**100.0**	**0**	**0**	**7**	**7**	**100.0**

Bold-Summary of drug administration per antimicrobial group, the corresponding active ingredients are indicated below. 0 observed zero i.e. not administered during the period of study.

### Amount of Antimicrobial active ingredients used for dogs and cats at the Veterinary Teaching Hospital (A and B) from 2019 to 2021

**Figure 3:**
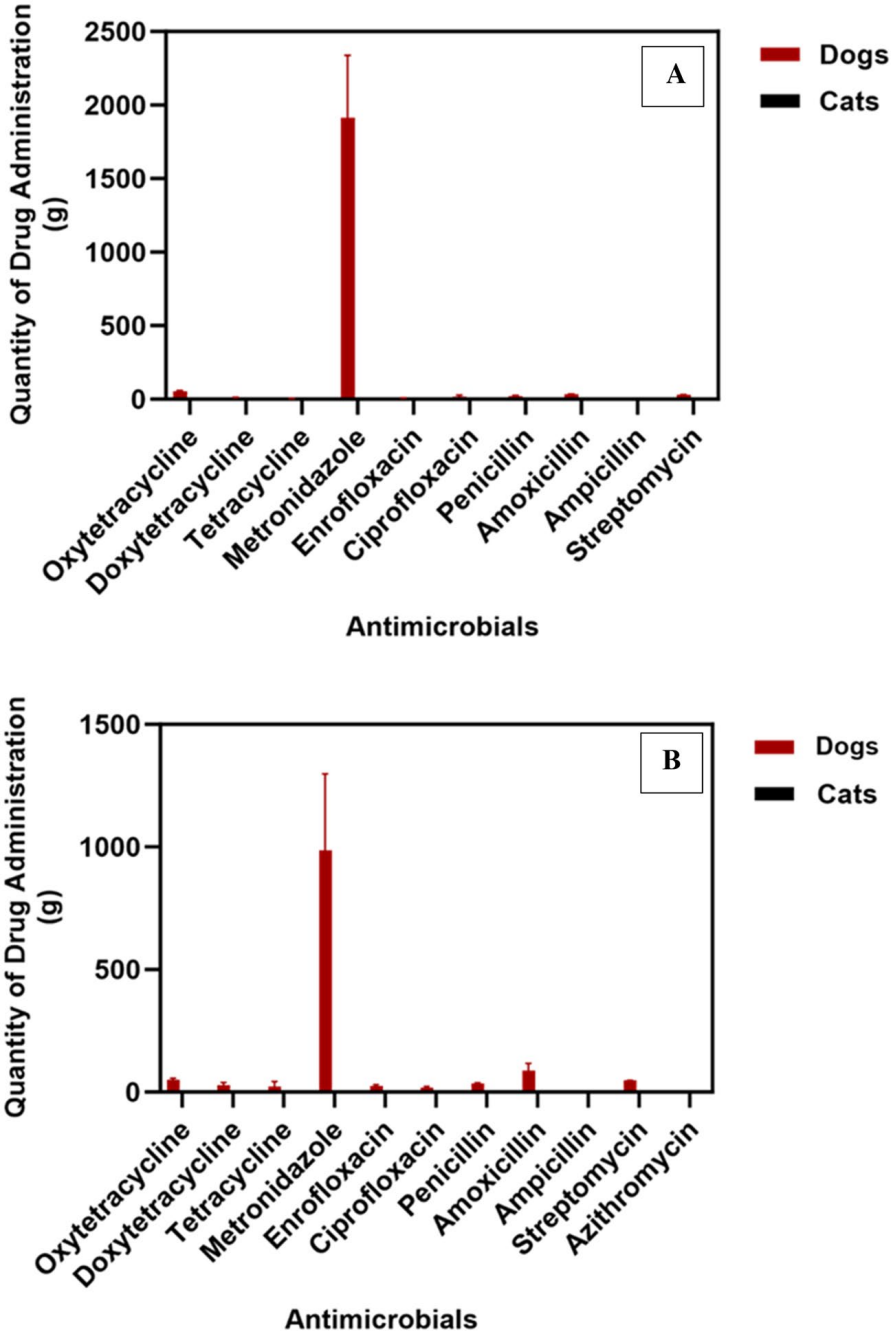
The mean quantity of antimicrobial ingredients administered at (**A**) VTH-A, and (**B**) VTH-B. Error bars are SEM of data sets for three years.

The documented amount of AIs used from 2019 to 2021 was 10.1 kg (A, 6.2 kg and B, 3.9 kg respectively, Fig. 3A and B). The quantity of antimicrobials used at VTH-A and B are presented in Tables 4 and 5. The largest amount of antimicrobial used was in dogs of which metronidazole accounted for (92.0%, 5.7 kg) and 75.9% (3.0 kg) at A and B respectively. Conversely, the lowest amount of AIs was administered to cats during the study period (0.0173 kg). The quantity of antimicrobials administered was similar (P = 0.15) at VTH-A and B, while the quantity of metronidazole administered in dogs was significantly higher (P < 0.0001) than in other AIs.

**Table 4:** Documented amount of antimicrobial active ingredients used in dogs and cats in 2019, 2020, and 2021 at the Veterinary Teaching Hospital-A.

	Dogs					Cats				
	2019 Amount (g)	2020 Amount (g)	2021 Amount (g)	Total amount (g)	Total amount (%)	2019 Amount (g)	2020 Amount (g)	2021 Amount (g)	Total amount (g)	Total amount (%)
Tetracyclines	**80.6**	**41.5**	**65.5**	**187.6**	**3.0**	**0.3**	**0.0**	**0.0**	**0.3**	**1.6**
Oxytetracycline	53.6	39.2	63.5	156.3	2.5	0.3	0.0	0.0	0.3	1.6
Doxycycline	19.4	2.3	2.0	23.7	0.4	0.0	0.0	0.0	0.0	0.0
Tetracycline	7.6	0.0	0.0	7.6	0.1	0.0	0.0	0.0	0.0	0.0
Penicillins	**36.8**	**56.8**	**66.1**	**159.7**	**2.6**	**0.0**	**0.1**	**5.0**	**5.1**	**29.5**
Penicillin	15.6	20.4	29.0	65.0	1.0	0.0	0.0	5.0	5.0	28.9
Amoxicillin	21.2	36.4	37.1	94.6	1.5	0.0	0.1	0.0	0.1	0.6
Ampicillin	0.0	0.04	0.0	0.04	0.1	0.0	0.0	0.0	0.0	0.0
Aminoglycosides	**19.5**	**25.5**	**36.9**	**81.9**	**1.3**	**0.0**	**0.0**	**6.3**	**6.3**	**36.1**
Streptomycin	19.5	25.5	36.9	81.9	1.3	0.0	0.0	6.3	6.3	36.1
Macrolides	**0.0**	**0.0**	**0.0**	**0.0**	**0.0**	**0.0**	**0.0**	**0.0**	**0.0**	**0.0**
Azithromycin	0.0	0.0	0.0	0.0	0.0	0.0	0.0	0.0	0.0	0.0
Imidazoles	**1540.7**	**2760.4**	**1445.1**	**5746.2**	**92.0**	**4.1**	**1.4**	**0.0**	**5.5**	**31.8**
Metronidazole	1540.7	2760.4	1445.1	5746.2	92.0	4.1	1.4	0.0	5.5	31.8
Quinolones	**45.8**	**6.4**	**15.6**	**67.8**	**1.1**	**0.0**	**0.0**	**0.3**	**0.3**	**1.7**
Enrofloxacin	7.3	1.1	10.6	19.0	0.3	0.0	0.0	0.0	0.0	0.0
Ciprofloxacin	38.5	5.3	5.0	48.8	0.8	0.0	0.0	0.3	0.3	1.7
Total	**1723.4**	**2890.6**	**1629.1**	**6243.1**	**100.0**	**4.2**	**1.5**	**11.6**	**17.3**	**100.0**

Significant values are in [bold].

**Table 5:** Documented amount of antimicrobial active ingredients used in dogs and cats in 2019, 2020, and 2021 at the annex, VTH-B.

	Dogs					Cats				
	2019 Amount (g)	2020 Amount (g)	2021 Amount (g)	Total amount (g)	Total amount (%)	2019 Amount (g)	2020 Amount (g)	2021 Amount (g)	Total amount (g)	Total amount (%)
Tetracyclines	**126.8**	**57.3**	**116.7**	**300.8**	**7.7**	**0.0**	**0.0**	**0.0**	**0.0**	**0.0**
Oxytetracycline	48.8	37.2	63.2	149.2	3.8	0.0	0.0	0.0	0.0	0.0
Doxycycline	15.3	17.6	50.3	83.2	2.1	0.0	0.0	0.0	0.0	0.0
Tetracycline	62.7	2.5	3.2	68.4	1.8	0.0	0.0	0.0	0.0	0.0
Penicillins	**98.9**	**91.7**	**175.7**	**366.3**	**9.4**	**0.0**	**0.0**	**0.0**	**0.3**	**0.3**
Penicillin	40.8	35.8	28.7	105.3	2.7	0.0	0.0	0.0	0.0	0.0
Amoxicillin	58.1	55.9	147.0	261.0	6.7	0.0	0.0	0.3	0.3	0.3
Ampicillin	0.0	0.0	0.0	0.0	0.0	0.0	0.0	0.0	0.0	0.0
Aminoglycosides	**50.5**	**44.8**	**44.1**	**139.4**	**3.6**	**0.0**	**0.0**	**0.0**	**0.0**	**0.0**
Streptomycin	50.5	44.8	44.1	139.4	3.6	0.0	0.0	0.0	0.0	0.0
Macrolides	**0.5**	**0.6**	**0.0**	**1.1**	**0.0**	**0.0**	**0.0**	**0.0**	**0.0**	**0.0**
Azithromycin	0.5	0.6	0.0	1.1	0.0	0.0	0.0	0.0	0.0	0.0
Imidazoles	**362.6**	**1264.4**	**1330.9**	**2957.9**	**75.9**	**0.0**	**0.0**	**0.0**	**0.0**	**0.0**
Metronidazole	362.6	1264.4	1330.9	2957.9	75.9	0.0	0.0	0.0	0.0	0.0
Quinolones	**41.9**	**38.9**	**49.5**	**130.3**	**3.3**	**0.0**	**0.0**	**0.0**	**0.0**	**0.0**
Enrofloxacin	34.9	21.9	21.6	78.4	2.0	0.0	0.0	0.0	0.0	0.0
Ciprofloxacin	7.0	17.0	27.9	51.9	1.3	0.0	0.0	0.0	0.0	0.0
Total	**681.1**	**1497.6**	**1716.9**	**3895.6**	**100.0**	**0.0**	**0.0**	**0.3**	**0.3**	**100.0**

Significant values are in [bold].

### Antimicrobial classification based on the risk to human health and prioritization for antibiotic stewardship according to the World Health Organization (WHO)

**Figure 4:**
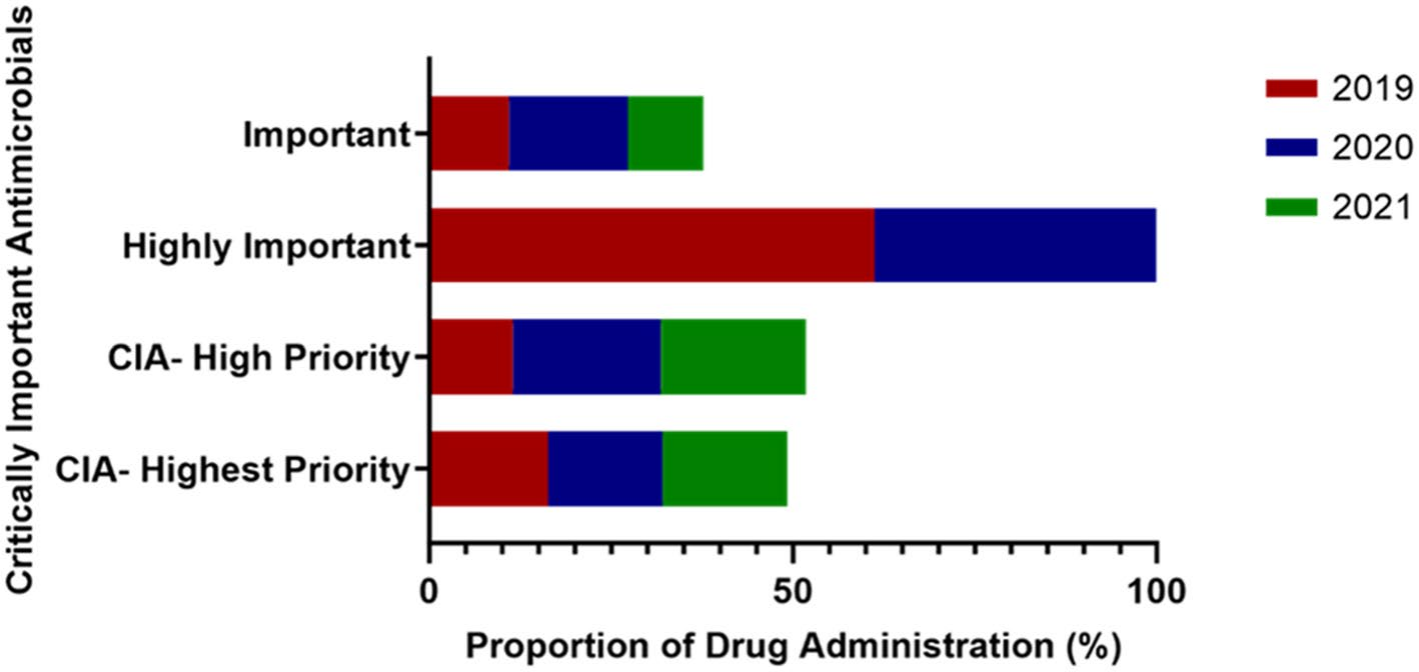
Proportion of documented antimicrobial drug administrations at the Veterinary Teaching Hospital and its annex from 2019 to 2021 and classification according to the WHO critically important antimicrobials (CIA) and AWaRe (Access, Watch, and Reserve).

Antimicrobials documented were medically important antimicrobials. AIs were classified as CIAs with highest priority (16.5%), high priority (17.5%), highly important (53.7%), and important (12.2%; Fig. 4). Five out of the AIs fell under the Watch group and included all the CIA's highest priority agents namely enrofloxacin, ciprofloxacin, and azithromycin (Table 6).

**Table 6:** Classification of Antimicrobial class and active ingredients according to World Health Organization (WHO) critically important antimicrobials in humans, and AWaRE groups (Access, Watch, Reserve).

Antimicrobial group	Active ingredient	WHO classification (Critically important in Humans)	Access	Watch	Reserve
Quinolones	Enrofloxacin	CIA*–highest priority	-	√	-
	Ciprofloxacin	CIA*–highest priority	-	√	-
Macrolides	Azithromycin	CIA*–highest priority	-	√	-
Penicillins	Amoxicillin	CIA*–high priority	√	-	-
	Ampicillin	CIA*–high priority	√	-	-
	Penicillin (narrow spectrum)	Highly important	√	-	-
Aminoglycosides	Streptomycin	CIA*–high priority	-	√	-
Tetracyclines	Tetracycline	Highly important	√	-	-
	Doxycycline	Highly important	√	-	-
	Oxytetracycline	Highly important	-	√	-
Nitroimidazoles	Metronidazole	Important	√	-	-

*CIA, critically important antibiotics in human medicine; Access, this group includes antibiotics that have activity against a wide range of commonly encountered susceptible pathogens and show lower resistance potential than antibiotics in the other groups; Watch, group includes antibiotic classes that have higher resistance potential and includes most of the CIA highest priority agents; Reserve, antimicrobial group that should be reserved for treatment of confirmed or suspected infections due to multi-drug-resistant organisms (last resort when all alternatives have failed).

### In-depth interview responses

Three APCs provided consent to be interviewed. The themes of discussion and responses are presented in Table 7.

**Table 7:** Responses and summary of the In-depth Interview (IDI) held among clinicians at the VTH and Its annex.

Items discussed	Summary	Responses transcribed verbatim
Theme 1: Perceptions about antimicrobial use practices, prescribing, abuse, and challenges		
Drivers of choice of the antimicrobials used in the VTH	APCs reported that the choice of antimicrobials is fundamentally determined by the case presentation, formal knowledge and experiences, clients’ willingness to comply with the prescribed antibiotic therapy regimen, availability of the drug and cost, and laboratory results where available	“*Antimicrobial choice is dependent on the nature of the case. Different cases have different protocols to follow in handling the cases. Drug availability and cost, availability of the clients to persist in presenting their pets to complete antibiotic therapy regime, temperament of the patient are also factors*” “*The clinical signs, those are the evidence before me, and lab results, if available from the labs. If I strongly feel that I should use a particular antibiotic, then I use it. Then the availability of the drug and the client may likely be influenced. So if we do not have a particular drug then we may need to switch to another drug. We work based on clinical signs, severity of infection, and laboratory diagnosis. The years of experience and the knowledge of the use of the drug also determines our decision on what antibiotics to use*”
The rationale behind the high level of oxytetracycline and route (I/V) of use in the practice	APCs provided reasons for the higher administration of oxytetracycline compared with other active ingredients. According to the protocol in the practice, oxytetracycline is the first line of treatment for blood parasitism, especially babesiosis (aside from bacterial infections), which is an endemic blood parasite infection in animals in Nigeria	*“It is because oxytet is also used to treat protozoal infections (Babesiosis) apart from bacterial infections. When we do blood screening or observe tick infestations, then we use oxytetracycline”* *“Although Berenil is an alternative drug and even the first choice of drug to treat Babesiosis in literature but oxytetracycline is preferred to Berenil because of the neurotoxic and hepatotoxic effects of Berenil*” “*Honestly, I would love to say that Berenil is actually the first drug of choice for babesiosis on paper. It is not as if it is not effective, it is excellent but it causes liver toxicity. There are sometimes that it causes cerebral involvement. That is why we don’t want to go there. It has a very thin, I mean low margin of safety whereas oxy has a very wide margin of safety. So we would rather take the one with a wide margin of safety than take the one that has low margin of safety and we have more mortalities. Not everybody is familiar with the use of Berenil*” *“I and another researcher had a paper on diminazene toxicity in dogs, and this is what opened our eyes to see that we can’t keep on using Berenil, we have to check other safer options. So that is why I said actually in book, Berenil is the drug of choice but in practice, it is not”* “*In my own opinion, oxytetracyclines have been working not only for babesiosis but for other blood parasites. We have also used doxy especially when it’s in a combination of babesiosis and ehrlichiosis*”
Reasons why higher quantities of metronidazole are used compared with other antimicrobials in the hospital and its annex	APCs said that the large quantity of metronidazole consumption may be associated with its dosage range, safety, and severity of disease	*“Metronidazole has a higher dosage range of 10–30 mg/kg compared to Oxytet that has dosage range of 5–10mg/kg”* “*Severity of the case also influences the clinician’s choice to use the higher dose rate of the drug*” *“Three factors affect the high dose of met- severity of infection, weight of animal, and the drug dosage. If the severity is low, they use low dosage range (10mg/kg) and if severity is high someone will take the higher dosage (30mg/kg). If we have more of them having severe infection, the clinicians will naturally take 30 mg/kg. Also, the frequency of administration of Metronidazole is a factor. It is administered more frequently like bi-daily”*
Written standard operating guidelines or antimicrobial use policy for CAs in the practice	Used to have documents available	*“We have it in our head. It is not written or documented”* *“We used to have them documented”*
Antimicrobial administration and duration	APCs indicated antimicrobials are used according to the manufacturer’s guideline	“*Usually 5–7 days*^*”*^
Clients’ compliance to antimicrobial therapy plan for their pets?	Clinicians indicate that they follow up with clients to ensure compliance with antimicrobial therapy plans for their pets	**“***We do follow-up few times by phones. We usually start all over again if clients break the therapy and then follow up to ensure compliance*”
Strengths of the practice that impact antimicrobial use	A few strengths were identified by participating clinicians. These included having well-trained veterinary personnel, who ensure that proper diagnosis is carried out based on their good knowledge and experiences of disease presentations, and laboratory confirmation. The practice carries out patient-centered approach investigations and treatments	**Strengths** *“We have good experience and good knowledge about the cases presented and also have good knowledge of the antibiotics to be used”* “*We try as much as possible to do proper diagnosis. With proper diagnosis, then we go to the lab. When we have proper diagnosis with lab confirmation we corroborate this to inform the use of antibiotics. And that is one strength we have- we almost always want to have a reason for antibiotic use. We do not like to just bump into antibiotics just like that. We want to have a strong reason for antibiotic use*” “*Another thing is that we have good consultants that go through our cases and they advise us. And I think that has really helped us. For instance, recently based on consultant advice, we had to shift from the use of other antibiotics to now cephalosporins, especially for the treatment of parvo*” “*We have outsourced labs that we send samples to for proper diagnosis*” “*We take each case individually even if the animals come from the same place (we do patient-centered approach)*”
Gaps identified that impact antimicrobial use in CAs in Nigeria	Several gaps that may promote indiscriminate use of antimicrobials in CAs were identified by clinicians. These included limited funding for antimicrobial sensitivity tests, inadequate veterinary laboratories, poor funding, quackery, lack of structure for capacity building or on-job training, especially on antimicrobial stewardship	**Gaps** “*Sometimes antimicrobial sensitivity tests are not carried out before patient treatment, inadequate availability of veterinary laboratories and lack of proper funding*” “*We have many non-vets (Quacks). They are so many now in companion animals because everybody wants to be a breeder, and everyone is beginning to recognize that they can make a lot of money. Because of the high level of insecurity in the country, most families and homes want dogs so everybody has ordinarily woken up to become a vet doctor. So we are having serious issues with quacks. By the time the cases come they would have used all sorts of drugs that you can’t even imagine, they would have finished the kidneys. Worst still, there are some human doctors that tend to treat their pets themselves. They will tell you what they used and the reason why they used it*” “*There is the lack of capacity building among staff/Lack of on-job training for staff on antimicrobial stewardship*”. *Currently, there may not be staff training in some veterinary teaching hospitals”*
Indiscriminate use of antimicrobials in companion animals in Nigeria	Antimicrobials are indiscriminately used in CAs according to the APCs. They also indicated most especially by quacks and clients, especially dog breeders who self-administer drugs to their pets	“*Yes and it is most common among the breeders because they mostly self-treat their pets before presenting them to vets when the condition is not improving*”
Recommendations to combat or to reduce indiscriminate use of antimicrobials?	APCs recommendations towards combating indiscriminate use of antimicrobials are summarized and include target-specific training and awareness creation, control of quackery in the veterinary profession, and effective surveillance of antimicrobial use and handling	“*Creating more awareness about it and its consequence of leading to antimicrobial resistance to the public, curbing quackery, and regulating the sales and handling of antimicrobials*”
Theme 2: Infection control practices		
Infection control practices/policies documented	The teaching hospital has an infection control policy	“*Well, I would say, honestly, that I feel, I think we have*”
Theme 3: Awareness of the WHO—CIA and AWaRe groups		
Are you aware of the CIA in human medicine and the WHO-AWaRe classifications?	APCs reported they have heard of the WHO-CIA groups. But unaware of the AWaRe classification	“*Yes, we have heard about the WHO-CIA classification but no not aware of the AWaRe*”. “*We are hearing about this for the first time*”
Are you aware that some of the antimicrobials used in the VTH are under the AWaRe-watch group e.g. the quinolones?	Clinicians were unaware of antimicrobials classifications under the AWaRe-watch group	“*No*”
Recommendations for prioritizing antimicrobials in this group	APCs believe there is a need to intensify training of staff especially in responsible use of these antimicrobials. There should be a documented policy that will enhance the hospital's prioritized use	“*Well, like having policies that guide our drug use and everything that will help us to naturally work in prioritizing. If we have drug use policies and everything laid down these policies will work based on what you know, we use drugs from low to high. And actually, that is what we try to do as much as possible so that we don’t start from antibiotics that are high up the ladder. We try to start from the low ones and then if we now have issues, we switch to medium or higher antibiotics*”
How about making antimicrobial sensitivity testing mandatory?	The clinicians perceive this will be difficult due to limited funding for such procedures and on the part of the clients who may not be ready to pay for such services. Also, the overuse and abuse of antimicrobials by non-vets make AST very unhelpful especially where resistance (100%) to all antimicrobials is reported	“*That will be a little bit difficult because of funds. Then this means the clients have to pay anytime we do that. So, if we have to do AST for every patient, some of these clients themselves even abuse these drugs so even if we have to do AST, it may not really help us*” “*Yes, AST will help us know that okay certain drugs have been abused by the client but some AST that has been done, we have had results where we’ve seen resistance all through. When we have resistance all through, and the dog is coming to us for the first time, we know that either quacks have been treating or they have been treating themselves. Where do you start from when all antibiotics are highly resistant*?” “*We may not be able to enforce par say but as much as possible we can encourage our clients. So that the learned ones, we can start with them and tell them about the need for AST. We could create awareness on AMR*”

#### Theme 1: perceptions about antimicrobial use, prescribing drivers, and challenges

The clinicians who participated in the in-depth interview reported that the choice of antimicrobials is fundamentally determined by the case presentation, formal knowledge and experiences, clients’ willingness to persist in presenting their pets to complete antibiotic therapy regime, availability of the drugs and cost, and laboratory results if available. Also, APCs provided the reason for the higher administrations and quantity of oxytetracycline and metronidazole respectively compared with other active ingredients. Oxytetracycline is considered the first line of treatment for blood parasitism especially babesiosis (aside from bacterial infections), which is an endemic blood parasitic infection in animals in Nigeria. For metronidazole, the higher consumption was attributed to APC's preference to treat acute gastroenteritis, the dosage range, the safety of the drug, and the severity of the case presented. No documentation of antimicrobial policy or standardized treatment protocols was available.

A few strengths that impact the discriminate use of antimicrobials were identified by participating clinicians. These included having well-trained veterinary personnel, who ensure that proper diagnosis is carried out based on their good knowledge and experiences of disease presentation, and laboratory confirmation. The practice carries out patient-centered approach investigations and treatments as well.

Several gaps identified as promoting indiscriminate use of antimicrobials in CAs in Nigeria were discussed and reported. Factors such as lack of antimicrobial sensitivity tests and materials, inadequate veterinary laboratories, poor funding, quackery, and lack of structure for capacity building or on-the-job training especially on antimicrobial stewardship were discussed. APCs agreed antimicrobials are indiscriminately used in CAs most especially as a result of quackery. Clients (especially dog breeders) are reported to self-administer drugs to their pets promoting AMR in animals. APCs recommendations to combat indiscriminate use of antimicrobials included target-specific training and awareness creation, control of quackery in the veterinary profession, effective surveillance of AMU, prescriptions, and drug handling.

#### Theme 2: infection control policy/practices

The teaching hospital has infection control policies and practices but not officially documented or available to workers.

#### Theme 3: awareness about WHO-CIA and AWaRe

APCs were aware of the WHO-CIA groups and unaware of AWaRe classifications of antimicrobials of CIA in human medicine. APCs agree that there is a need to amplify efforts in the training of staff especially in the responsible use of these antimicrobials. In the clinicians’ opinion making antimicrobial susceptibility tests mandatory in companion animal practice may not be practicable due to limited funding for such procedures, and clients’ inability to pay for such services. The APCs pointed out that overuse and misuse of antimicrobials by non-vets especially quacks make the Antimicrobial Susceptibility Test (AST) unhelpful especially where resistance (100%) to all antimicrobials is reported.

## Discussion

The indiscriminate use of antimicrobials and the consequent impact on AMR in livestock animals have received more attention and monitoring by public health agencies than in CAs. Many countries still lack regulations on AMU in CAs and recommendations for responsible and prudent use, especially in African countries. Although antimicrobial quantity and misuse may be lesser in CAs, it is still crucial to monitor and report AMU considering the potential of dogs and cats to cross-transfer resistant microbes to humans through close-contact”. Furthermore, we must preserve and prioritize antimicrobial treatment possibilities as CAs share some classes of antimicrobials including medically important drugs with humans.

Presently, only a few studies have provided information about AMU, the classes, active ingredients, prescribing patterns, and quantity in companion animals^17,20–22^. In Nigeria, the systematic collection and evaluation of AMU data in companion animals are unavailable. There is no current data that demonstrates how much of these drugs have been used for dogs and cats in Nigeria. Hence, from a clinical context, we investigated for the first time qualitative and quantitative indices to describe different ACs, AIs, and quantity usage in dogs and cats presented at a VTH in Nigeria from 2019 to 2021.

The demographic characteristics of pets in the country indicated that dogs are kept as household pets more than cats are in Nigerian culture. This finding was in line with studies conducted on occupational hazards among veterinary students in southwest Nigeria who reported more contact with dogs than cats^23^. Cats are rarely kept as pets in Nigerian homes because of cultural and traditional myths that associate “terrible luck” with them^23^. Furthermore, the Alsatian/German Shepherd is the most common breed of dog reported to have visited the veterinary teaching hospital from 2019 to 2021. This finding is similar to Adekoya et al.^16^ who documented Alsatians (German Shepherd Dogs) as the most presented breeds of dogs at the veterinary teaching hospital. The result is not surprising because Alsatian/German shepherd is the most popular breed of dogs in Nigeria. This breed is loved for its strength, intelligence, and ability to be obedient and is often employed for security and protection by many families and individuals in the country.

The overall number of antimicrobial administrations was estimated and observed to be lower than reports from other recent studies conducted in Germany^17,18^, Denmark^24,25^, Italy^20^ and Japan^22^*.* The lower drug administrations reported in this study may be attributed to environmental and socio-economic variables impacting dog keeping and the number of dog owners in the country (estimated at about 10 million) as compared with the US (90 million), Europe (92.9 million), or China (136 million).

The main antimicrobial drugs used belonged to the classes of Tetracyclines (oxytetracycline, tetracycline, and doxycycline), Penicillin (amoxicillin and ampicillin), Quinolones (enrofloxacin and ciprofloxacin) and Nitroimidazoles (metronidazole). Presently, there are limited studies on AMU in companion animals in West Africa including Nigeria. However, our results were corroborated by a recent retrospective study, which investigated the trends in the clinical use and misuse of antibiotics in animals (companion animals, ruminants, and wildlife) at a veterinary hospital in Nigeria from 2013 to 2017^26^. The authors documented penicillin, streptomycin, oxytetracycline, gentamicin, and sulphadimidine as the most common antibiotics used. Furthermore, the frequent use of oxytetracycline was observed to increase during the study period. In another study conducted in Chile, penicillins, cephalosporins, quinolones, and tetracyclines were commonly prescribed drugs for Chilean CAs^21^. On the contrary, other studies^18,20,24,27^ showed that penicillins with extended-spectrum/beta-lactamase inhibitors, 1st and 2nd generation cephalosporins were frequently prescribed in CAs in the UK and EU. Similarly, in Japan, the most common were 1st generation cephalosporin and extended-spectrum penicillins^22^. Due to the high cost and unavailability of cephalosporins, cheaper AMs such as previously described are presently and frequently prescribed in CAs at the veterinary hospital. Furthermore, the high usage/ administration of oxytetracyclines may be attributed to its broad spectrum mode of action against Gram-positive and Gram-negative bacterial infections as well as for the treatment of canine babesiosis (an endemic blood-borne parasitic animal disease in Nigeria). These were corroborated by clinicians during the IDI who indicated that the high oxytet administration was due to its use in the treatment of blood parasite infections. Data on diseases that informed the type of antimicrobials prescribed for the treatment of companion animal diseases was not collected making this difficult to support why oxytetracycline was most prescribed at the VTH. It is likely the prescription of oxytetracycline could be linked to the treatment of babesiosis, which is the first line of management for the blood parasitic infection at the VTH. Other non-antibiotic therapeutic options for babesiosis exist and are strongly recommended. On the other hand, tetracyclines are used commonly in food animals in developed countries^22^, which could be a reflection of the difference between policies of antimicrobial drug use for food animals and policies for CAs.

The quantity of metronidazole used at the VTH was the highest compared with other AMs. Metronidazole (Flagyl®) is an antibacterial and antiprotozoal agent used in the treatment of certain anaerobic bacterial and protozoal infections. This drug has been in use since 1950. It inhibits several diarrheic agents making it many veterinarians' choice for diarrhea in general even for nonspecific diarrhea^9^. But in some cases, it may not be the best prescription based on several studies that have shown metronidazole does not address Giardia infection, inflammatory bowel disease, or acute diarrhea in dogs^28,29^. Current evidence-based research suggests that metronidazole is much less effective for some gastrointestinal conditions than previously thought and could make diarrhea worse by altering the intestinal mucus. It has been linked to causing unhealthy long-term changes in a pet’s gut microbiome^30,31^. A recent nonrandomized controlled study showed metronidazole significantly changed the gut microbiome composition, and decreased important beneficial bacteria, such as *Fusobacterial* (one of the dominant phyla of bacteria in the gut microbiomes of dogs and cats) that did not fully resolve 4 weeks post metronidazole treatment. The study also observed that it reduced overall richness (the number of different bacterial species in the gut microbiota) and caused fecal dysbiosis^31^. Alternative options to metronidazole and a more cautious approach to prescribing this antimicrobial to dogs are now being recommended^31^. Alternative approaches to metronidazole recommended in dogs and cats may include Rifaximin, a synthetic drug, that is as efficient as metronidazole in treating gastroenteritis,the use of probiotics; and natural options such as Diagel^32^.

The frequent routes of antimicrobial administration at the VTH were the intravenous (IV) and intramuscular (IM) site (especially for oxytetracyclines and enrofloxacin), and less oral (tetracycline, metronidazole, and amoxicillin) or intraocular (ciprofloxacin) However, this contradicts findings from other studies conducted in the United Kingdom^33^, Denmark^25^, and other European countries such as Finland ^34^, Norway^35^, Sweden^36^, Italy^20^ and the Netherlands^37^, which have shown oral route of AMs administrations prevailed over those for parenteral in dogs and cats. Drug administration via the oral route may be common and more attractive because it is non-invasive, painless, easy to perform though an essential skill requirement for all veterinarians, and well tolerated by patients^38^. Also, variation in antimicrobial protocol or policies across countries, or veterinary practices may contribute to this route being preferred. On the other hand, the efficacy, ease, and frequency of administration may support the use of IV oxytetracycline at the VTH in most cases. Furthermore, oral oxytetracycline is administered 3 times daily for 5–7 days in capsules and not all pet owners have the skill to administer oral medications. However, the success rate over the years has been good with IV oxytet based on clinicians’ experiences at the VTH.

For the prescription diversity (PD) finding, our study described higher PD values for dogs than cats. Current research conducted in Germany^17^ also corroborated our report, whichdocumented a significant increase in PD for AMs in dogs compared with cats. The PDs estimated were lower than those reported by Schnepf et al.^17^ and may be attributed to the frequency of dog and cat visits and the antimicrobial prescription protocols of the various veterinary care facilities.

For the critically important antimicrobials, drugs with the highest priority such as quinolones (e.g. enrofloxacin) were the most prescribed and the least being the macrolides (azithromycin). Ampicillin, which belongs to the CIA high priority was most administered. No drugs under the reserve group (last resort antimicrobials when all alternatives have failed) are used in CAs at the VTH unlike in the developed countries where fluoroquinolones and cephalosporins are mostly prescribed to treat infections^17,20,39,40^.

The researched veterinary care is a multispecialty university teaching hospital in Nigeria, and the present study was a small-scale project describing antibiotic use based on data extracted from the clinical or prescribing records. Though the study addressed AMU in companion animals for a period of three years, the study results may not be representative or generalizable to the other veterinary hospitals and clinics in the country. However, the work has created the first picture of qualitative and quantitative indices of antibiotic use in Nigerian CAs. Also, the study has generated data that could serve as a baseline for AMU negotiations, and the development of guidelines and strategies to promote antimicrobial stewardship in CAs in Nigeria. Another limitation is that it omitted critical information on pet diseases and microbiological laboratory tests performed, which made it difficult to link the motives for antimicrobial choice by clinicians. Further work may be needed to expand the period of evaluation of AMU retrospectively, describe common companion animal diseases, and the rationale for antimicrobial therapy in Nigeria.

In light of our result, the qualitative and quantitative assessments of antimicrobial usage in CAs showed that the most frequently used antimicrobial was oxytetracycline, while metronidazole had the highest administered amount. There was diversity in the prescription pattern of AMs especially in dogs. However, this may not be representative of the larger population of veterinary practices in the country. Hence, justifying the need for a broader national-based study to assess AMU in CAs in Nigeria. It is crucial to develop programmes or frameworks for the effective monitoring of antimicrobial prescriptions and usage/resistance in CAs in the country, and a national reporting system for veterinary practitioners which may contain detailed information, such as animal species, the reason for prescription, and dosage on each prescription. Also, there is the need to join efforts to control AMR development and focus on performing proper diagnosis, increasing the use of standardized microbiological laboratory tests to support diagnosis and appropriate treatment, developing antimicrobial protocol and infection control, creating awareness of AMU and AMR among stakeholders, and promoting staff training on antimicrobial stewardship within veterinary care facilities in Nigeria.

## Data Availability

The datasets generated during and/or analysed during the current study are available from the corresponding author on reasonable request.

## Abbreviations

ACs: Antimicrobial classes
ADA: Administered daily amount
AIs: Active ingredients
AAD: Antimicrobial administration
AMR: Antimicrobial resistance
AMs: Antimicrobials
AMS: Antimicrobial Stewardship
AMU: Antimicrobial use/usage
APC: Antimicrobial Prescribing Clinicians
AWaRe: Access, watch, reserve
AST: Antimicrobial susceptibility test
CAs: Companion animals
CIA: Critically important antibiotics
IDI: In-depth interview
TAD: Total administrations documented
PD: Prescription diversity
VTH: Veterinary Teaching Hospital
WHO: World Health Organisation
